# Comparing Artificial Intelligence and Senior Residents in Oral Lesion Diagnosis: A Comparative Study

**DOI:** 10.7759/cureus.51584

**Published:** 2024-01-03

**Authors:** Hamad Albagieh, Zaid O Alzeer, Osama N Alasmari, Abdullah A Alkadhi, Abdulaziz N Naitah, Khaled F Almasaad, Turki S Alshahrani, Khalid S Alshahrani, Mohammed I Almahmoud

**Affiliations:** 1 Oral Medicine, King Saud University, Riyadh, SAU; 2 Dentistry, College of Dentistry, King Saud University, Riyadh, SAU; 3 College of Dentistry, Dental University Hospital/King Saud University, Riyadh, SAU

**Keywords:** test, llm, oral diagonisis, chatgpt 3.5, oral medicine, artificial intellingence in dentistry

## Abstract

Introduction: Artificial intelligence (AI) is a field of computer science that seeks to build intelligent machines that can carry out tasks that usually necessitate human intelligence. AI may help dentists with a variety of dental tasks, including clinical diagnosis and treatment planning. This study aims to compare the performance of AI and oral medicine residents in diagnosing different cases, providing treatment, and determining if it is reliable to assist them in their field of work.

Methods: The study conducted a comparative analysis of the responses from third- and fourth-year residents trained in Oral Medicine and Pathology at King Saud University, College of Dentistry. The residents were given a closed multiple-choice test consisting of 19 questions with four response options labeled A-D and one question with five response options labeled A-E. The test was administered via Google Forms, and each resident's response was stored electronically in an Excel sheet (Microsoft® Corp., Redmond, WA). The residents' answers were then compared to the responses generated by three major language models: OpenAI, Stablediffusion, and PopAI. The questions were inputted into the language models in the same format as the original test, and prior to each question, an artificial intelligence chat session was created to eliminate memory retention bias. The input was done on November 19, 2023, the same day the official multiple-choice test was administered. The study had a sample size of 20 residents trained in Oral Medicine and Pathology at King Saud University, College of Dentistry, consisting of both third-year and fourth-year residents.

Result: The responses of three large language models (LLM), including OpenAI, Stablediffusion, and PopAI, as well as the responses of 20 senior residents for 20 clinical cases about oral lesion diagnosis. There were no significant variations observed for the remaining questions in the responses to only two questions (10%). For the remaining questions, there were no significant differences. The median (IQR) score of LLMs was 50.0 (45.0 to 60.0), with a minimum of 40 (for stable diffusion) and a maximum of 70 (for OpenAI). The median (IQR) score of senior residents was 65.0 (55.0-75.0). The highest and lowest scores of residents were 40 and 90, respectively. There was no significant difference in the percent scores of residents and LLMs (p = 0.211). The agreement level was measured using the Kappa value. The agreement among senior dental residents was observed to be weak, with a Kappa value of 0.396. In contrast, the agreement among LLMs demonstrated a moderate level, with a Kappa value of 0.622, suggesting a more cohesive alignment in responses among the artificial intelligence models. When comparing residents' responses with those generated by different OpenAI models, including OpenAI, Stablediffusion, and PopAI, the agreement levels were consistently categorized as weak, with Kappa values of 0.402, 0.381, and 0.392, respectively.

Conclusion: What the current study reveals is that when comparing the response score, there is no significant difference, in contrast to the agreement analysis among the residents, which was low compared to the LLMs, in which it was high. Dentists should consider that AI is very beneficial in providing diagnosis and treatment and use it to assist them.

## Introduction

The term artificial intelligence (AI) was discovered in the 1950s, and it stands for the ability of machines to do tasks that have been done by humans [1]. It is also known as part of computer science and focuses on designing intelligent systems that exhibit characteristics associated with human manners and behavior [2]. On the other hand, we have machine learning, which is a subdivision of artificial intelligence in how the algorithms are inserted to learn statistical patterns and structural patterns in the data, which allows it to foretell the unseen data. Machine learning has a popular model, which is neural networks [1]. Deep learning is a field in machine learning where multilayered neural networks represent hierarchical features in data [2]. Deep learning is helpful with complex data like pictures, as they are capable of representing their features [1]. In this situation of the enormous amount of data needed for implementing machine learning, data can refer to many types of inputs, such as clinical pictures, radiographs, and patient symptoms [3].

Large language models (LLMs) are AI programs that are trained on enormous amounts of textual data [4]. They use generative mathematical models of the statistical distribution of tokens in a large number of public corpuses of human-generated texts [4]. These tokens include graphemes, words, individual characteristics, and punctuation marks [4]. LLMs could generate fluent and solid texts, answers, and translations [5]. Big datasets that cannot be searched, analyzed, understood, or stored using conventional data-processing techniques are referred to as "big data." Big data include information from wearable technology, social media, mobile applications, environmental and lifestyle factors, sociodemographic, and "omic" (genomics, metabolomics, proteomics) data, as well as information from platforms for precision medicine or standardized electronic health records (EHRs). Big data alone is insignificant, but when combined with AI to interpret the data and generate predictions or judgments, it has the potential to revolutionize the way clinical care is currently provided [6]. AI can assist dentists in many dental tasks, such as treatment planning and clinical diagnosis, as it can detect and assist with oral maxillofacial defects and abnormalities [2]. It could also be useful for education and act as a teaching tool by providing schoolwork guidance and tutoring students by simplifying dental information to make it clear and understandable [7]. AI applications have advanced due to the mass of data gathered and made available in the healthcare industry, as well as improvements in computer power. This has resulted in an exponential rise in publications on AI in healthcare, with over 10,000 records on PubMed in 2021 alone. This includes numerous reviews from various medical fields that examine how AI might improve the overall delivery of healthcare [8]. AI has been used in diagnosing and treatment planning in many fields of dentistry, like periodontology, orthodontics, endodontics, prosthodontics, and oral and maxillofacial surgery [9]. Moreover, Al has been applied to dental radiology to enhance picture interpretation. Digital radiography in two dimensions (2D) is made up of thousands of pixels. Within the grid, each pixel unit represents a different brightness level. The radiopacity-presenting pixel can be classified as either having a greater density structure or as metal. All programs "learn" to analyze the digital image based on these features [10].

AI has been useful in decision-making, and it can reduce human errors in diagnosing and reduce the stress on dentists in decision-making [3]. The primary obstacle is not technology per se, which is expanding, changing, and finding new applications quickly; rather, it is the legal system, which is obviously deficient in suitable rules as well as in certain areas of politics, ethics, and finance. The main concerns are therefore whether or not this technology is appropriate for healthcare in the first place [11]. AI systems could potentially prove useful in a replacement scenario. AI might be able to complete some activities more consistently, quickly, and reproducibly than humans, even if it is unlikely to completely replace human healthcare workers.

Healthcare providers may be able to handle more complex tasks, resulting in enhanced use of human capital, by automating low-complexity but labor- and time-intensive tasks [12]. This paper evaluates the performance of oral medicine residents in diagnosing different cases, compares it with different AI programs in various case-based scenarios and treatment planning, and sees its reliability in assisting the residents in diagnosing and making decisions.

## Materials and methods

An exam with 19 questions containing four labeled as (A-D) response options and one question with five labeled as (A-E) response options is formatted as a closed multiple-choice test created by a content area expert. The test was done in Google Forms, and the questionnaire can be found in the appendices section. Third-year and fourth-year residents of Oral Medicine and Pathology, trained at King Saud University, College of Dentistry, participated in this test. After they gave their approval and obtained consent to take part in the research, a certified consultant used a Google Form for each resident. All information was stored in an electronic sheath (Excell) as of November 2, 2023. The residents' answers are analyzed and compared to the responses from three major language models (OpenAI, Stablediffusion, and PopAI) as a comparative study.

Questions input and responses output

The questions were entered into three large language models, namely OpenAI, Stablediffusion, and popAI, in the same format as they were on the official multiple-choice test, and the version of ChatGPT used was 3.5. The multiple-choice response options were labeled A-D for questions with four options and A-E for questions with five options. Prior to the input of each question, an artificial intelligence chat session was created for each question in order to prevent memory retention bias. Three different AI engines were employed to minimize bias. Every question was entered precisely as it was seen on November 19, 2023, when the Progress Test was administered.

Sample size

Twenty residents in their third- and fourth-year training at King Saud University, College of Dentistry, went through the questionnaire.

Case materials

A consultant in oral medicine and pathology wrote the single correct answer to each of the 20 written clinical questions in this study, which featured a scenario involving oral or systemic disease. Senior residents were expected to correctly answer each clinical question, which included a typical presentation.

Statistical analysis

Statistical analysis was carried out using RStudio (R version 4.3.1, RStudio, Boston, MA). Frequencies and percentages are used to present categorical variables. Focusing on the clinical case questions, one was assigned for the correct response and 0 for the incorrect response. An overall knowledge score was computed by summing up the correct values of responses from residents and LLMs. For each subject (LLM or resident), the overall score ranged between 0 and 20, where higher scores indicated a higher knowledge level (higher frequencies of correct responses). To facilitate interpretation, we standardized these raw scores to a percent score. This was performed using the following formula: percent score = (raw score * 100)/20. We compared the performance of LLMs and residents by comparing the percent knowledge scores using a Wilcoxon rank sum test, and the scores were expressed as medians and interquartile ranges (IQRs). The differences between the responses of LLMs and residents were compared using Fisher's exact test. Response agreement analysis was carried out using Light's Kappa as a parameter of interrater agreement between different subjects.

Ethical approval

The Institutional Review Board (IRB) and Institutional Committee of Research Ethics of King Saud University in Riyadh, Saudi Arabia, approved the study for ethical considerations (permission number: E-23-8210). The 2013 revision of the 1975 Helsinki Declaration was followed while conducting the study. Prior to their enrollment, all participants provided their informed permission.

## Results

**Figure 1:**
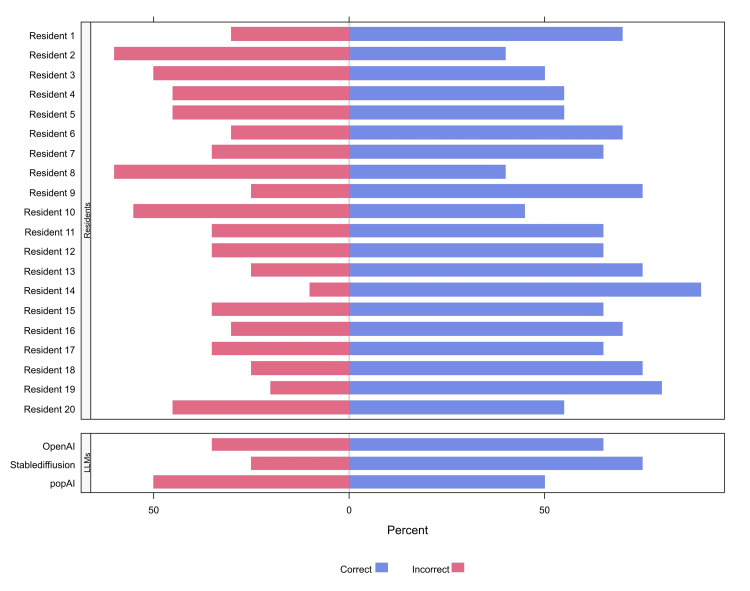
The proportions of correct answers for individual residents and each large language model LLMs: large language models

**Figure 2:**
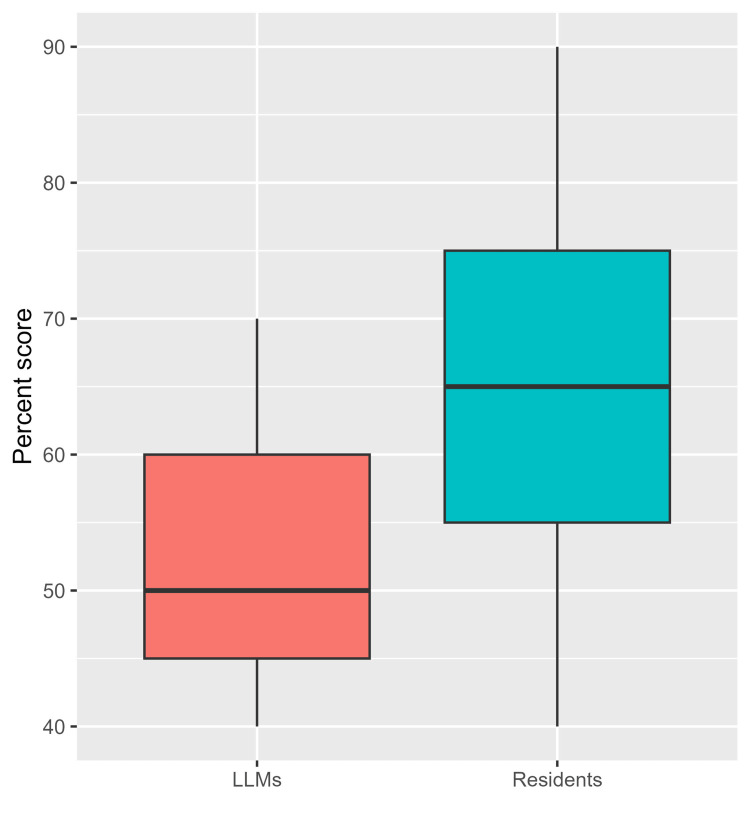
Boxplots depicting the percent knowledge score among residents (n=20) and large language models (n=3) LLMs: large language models

In the current study, the responses of three large language models were analyzed, including OpenAI, Stablediffusion, and PopAI, as well as the responses of 20 senior residents for 20 clinical cases of oral lesion diagnosis. Table 1 summarizes the responses to each individual question. Out of the 20 questions, there were significant differences in the responses to only two questions (10%). In a case involving the diagnosis of ectodermal dysplasia, none of the LLMs responded accurately (0.0%), and all of the models incorrectly identified the cases as xeroderma pigmentosum. On the other hand, 85.0% of senior dental residents have correctly identified the diagnosis (p = 0.011). Furthermore, the performance divergence was evident in the management of a large painful ulcer post-kidney transplant, where all three LLMs favored topical corticosteroid (100%), while 70.0% of senior dental residents correctly leaned towards intralesional corticosteroid injection (p = 0.022). For the remaining questions, there were no significant differences in the proportions of correct answers between LLMs and residents (Table 1). More details about the proportions of correct and incorrect answers for individual residents and LLMs are depicted in Figure 1. Based on the percent knowledge scores, the median (IQR) score of LLMs was 50.0 (45.0 to 60.0, Figure 1), with a minimum of 40 (for Stablediffusion) and a maximum of 70 (for OpenAI, Figure 2). The median (IQR) score of senior residents was 65.0 (55.0-75.0, Figure 1). The highest and lowest scores of residents were 40 and 90, respectively. There was no significant difference in the percent scores of residents and LLMs (p = 0.211).

**Table 1:** The responses of residents and large language models to the questions used in the current study LLMs: large language models, ALT: alanine transaminase, AST: aspartate aminotransferase, mmHg: millimeters of mercury, CBC: complete blood count, TMJ: temporomandibular joint. *An asterisk indicates a correct response. ^Regarding questions with high concern.

Questions	Choices	LLMs, N=3	Residents, N=20	p-value
A 50-year-old male sought a dental checkup and oral prophylaxis, noting a three-year interval since his last dentist visit. The patient, a daily smoker of 10 cigarettes, reported no other systemic diseases. Examination revealed painless, well-demarcated whitish plaques on the buccal mucosa and both retro-commissures of the lips, which did not detach upon rasping. The patient was uncertain about the duration of these lesions. The clinical features of the white lesions could be diagnosed as:	Pseudomembranous candidiasis	0 (0.0%)	0 (0.0%)	>0.999
	Erythematous candidiasis	0 (0.0%)	0 (0.0%)	
	Hyperplastic candidiasis*	3 (100.0%)	19 (95.0%)	
	Angular cheilitis	0 (0.0%)	1 (5.0%)	
	Herpes simplex virus	0 (0.0%)	0 (0.0%)	
A 55-year-old female patient with a history of type II diabetes mellitus presented to your clinic with a new-onset (3 months duration) solitary oral pigmented lesion measuring 1 cm × 1 cm on the lower lip. The lesion appears brown to dark brown in color, and its size has remained constant.	Perform a biopsy*	3 (100.0%)	12 (60.0%)	0.644
	Request a complete blood test	0 (0.0%)	2 (10.0%)	
	Follow up the case and do nothing	0 (0.0%)	6 (30.0%)	
	Take an X-ray to check for any amalgam remnants	0 (0.0%)	0 (0.0%)	
^A 21-year-old male patient presented at your clinic, and during the extra-oral examination, you noted fine, sparse hair, wrinkled, and hyperpigmented periocular skin. This patient is most likely experiencing:	Ectodermal dysplasia*	0 (0.0%)	17 (85.0%)	0.011
	Xeroderma pigmentosum	3 (100.0%)	3 (15.0%)	
	Darier disease	0 (0.0%)	0 (0.0%)	
	Peutz-Jeghers syndrome	0 (0.0%)	0 (0.0%)	
A 10-year-old boy, presenting with crusted lips and a history of fever, exhibits red blisters with white centers on his face, hands, and feet. This boy most probably has:	Hand-foot and mouth disease	3 (100.0%)	10 (50.0%)	0.339
	Erythema multiforme triggered by food	0 (0.0%)	0 (0.0%)	
	Erythema multiforme triggered by HPV infection	0 (0.0%)	1 (5.0%)	
	Erythema multiforme triggered by HSV infection*	0 (0.0%)	9 (45.0%)	
A 35-year-old female visited your clinic seeking restoration for her missing lower first molars, teeth #36 and #46. During history-taking, she mentioned a three-month complaint of psoriasis, treated with hydrocortisone for affected skin lesions. What oral manifestations would you expect to observe in her condition?	Fissure tongue	2 (66.7%)	3 (15.0%)	0.232
	Erythema migrans*	1 (33.3%)	16 (80.0%)	
	Hairy tongue	0 (0.0%)	1 (5.0%)	
	Scalloped tongue	0 (0.0%)	0 (0.0%)	
A 45-year-old female patient with a history of hepatitis C, hypertension, elevated ALT and AST, and blood pressure measured at 135/105 mmHg, presented to the oral medicine clinic with extensive ulcerations, including the buccal mucosa and anterior tongue. Additionally, the lower labial mucosa exhibited white lacy lines with intact mucosa. The most probable diagnosis would be:	Mucous membrane pemphigoid	1 (33.3%)	0 (0.0%)	0.130
	Erosive lichen planus*	2 (66.7%)	20 (100.0%)	
	Erythema multiforme	0 (0.0%)	0 (0.0%)	
	Pemphigus vulgaris	0 (0.0%)	0 (0.0%)	
A 42-year-old female patient attending the oral diagnosis course clinic exhibits claw-like fingers and a mouse face. Upon inquiry, she reveals that she has been diagnosed with:	Multiple sclerosis	0 (0.0%)	5 (25.0%)	>0.999
	Systemic lupus erythematosus	0 (0.0%)	0 (0.0%)	
	Systemic sclerosis*	3 (100.0%)	15 (75.0%)	
	Tuberous sclerosis	0 (0.0%)	0 (0.0%)	
A 55-year-old female patient, complaining of a toothache, reports a history of splenectomy due to splenomegaly five years ago. You order a complete blood count (CBC) to ensure everything is within normal limits. What would you expect to be elevated in the CBC report?	Red blood cells count	0 (0.0%)	10 (50.0%)	0.322
	Platelets count*	3 (100.0%)	9 (45.0%)	
	Neutrophils count	0 (0.0%)	1 (5.0%)	
	Eosinophils count	0 (0.0%)	0 (0.0%)	
In diagnostic anesthesia methods used to localize a source of pain, which of the following conditions would you NOT anticipate a positive response (significant decrease in pain) to a local anesthetic (both injection and topical)?	Atypical odontalgia*	2 (66.7%)	12 (60.0%)	>0.999
	Trigeminal neuralgia	1 (33.3%)	4 (20.0%)	
	Glossopharyngeal neuralgia	0 (0.0%)	2 (10.0%)	
	Traumatic neuroma	0 (0.0%)	2 (10.0%)	
^A 55-year-old female patient, who underwent a kidney transplant three months ago, presents at your clinic with a large painful ulcer in the buccal mucosa. What is the best treatment for this ulcer?	Topical Lidocaine gel	0 (0.0%)	3 (15.0%)	0.022
	Topical corticosteroid	3 (100.0%)	3 (15.0%)	
	Intralesional corticosteroid injection*	0 (0.0%)	14 (70.0%)	
	Systemic corticosteroid	0 (0.0%)	0 (0.0%)	
An elderly male patient with a history of Bechet’s disease with long-term steroid use. He came to your clinic to report experiencing pain and swelling on the left side of his face, along with a 2-week period of erythema on his skin. The pain aggravated during mealtime. An intraoral examination revealed pus discharge from the left Stenson's duct. What diagnosis of the following in the top of the list?	Sjögren's syndrome	0 (0.0%)	1 (5.0%)	>0.999
	Lymphoma	0 (0.0%)	0 (0.0%)	
	Bacterial sialadenitis*	3 (100.0%)	19 (95.0%)	
	Mucoepidermoid carcinoma	0 (0.0%)	0 (0.0%)	
Your clinic was visited by a 23-year-old ex-ballet dancer who reported a loud clicking sound on both sides of her TMJs without experiencing any discomfort. The opening of her mouth is 55 mm, with a slight deviation to the right then to the left starting suddenly 16 months ago. Even after mandibular advancement, the examination revealed a clicking sound. The most likely diagnosis in this case is:	Disc displacement with reduction	1 (33.3%)	10 (50.0%)	0.404
	Disc displacement without reduction	2 (66.7%)	4 (20.0%)	
	Hypermobility/subluxation of the TMJs*	0 (0.0%)	6 (30.0%)	
	Dislocation of the TMJs	0 (0.0%)	0 (0.0%)	
A 41-year-old female patient who was well-nourished came forward with a main complaint of painful and limited mouth opening. When she opens, her mouth moves towards the right side. One week ago, she reported experiencing a trauma from her teenage son. What is the most likely explanation for this case?	Disc displacement with reduction in the left TMJ	0 (0.0%)	2 (10.0%)	0.743
	Chronic disc displacement without reduction in the right TMJ	0 (0.0%)	2 (10.0%)	
	Acute disc displacement without reduction in the right TMJ*	2 (66.7%)	13 (65.0%)	
	Acute disc displacement without reduction in the left TMJ	1 (33.3%)	3 (15.0%)	
A 31-year-old woman has been reported discomfort in her right cheek for the last two weeks. Vertical opening and excursions are normal. She has a feeling that her bite is off and it hurts when she attempts to bring her teeth together. Her examination revealed a posterior open bite on the right side and premature contact in the left anterior canine area. What is the most likely diagnosis?	Acute disc displacement of the right TMJ	2 (66.7%)	6 (30.0%)	0.81
	Capsulitis of the right TMJ	0 (0.0%)	6 (30.0%)	
	Acute disc displacement of the left TMJ	0 (0.0%)	2 (10.0%)	
	Retrodiscitis of the left TMJ*	1 (33.3%)	6 (30.0%)	
A 35-year-old female teacher came to your clinic complaining of severe pain on the left side of her jaw that started three months ago. Systemic osteoarthritis was a significant part of her medical history, and she was diagnosed with it last year. Her pain is intense and has a dull aching quality. The pain is making it difficult for her to open her mouth, even with paracetamol. The best way to control the pain in this case is achieved by?	Using non-steroidal anti-inflammatory drugs	3 (100.0%)	15 (75.0%)	>0.999
	Increasing the paracetamol dose	0 (0.0%)	0 (0.0%)	
	Starting antibiotic course	0 (0.0%)	0 (0.0%)	
	Giving corticosteroid injections*	0 (0.0%)	5 (25.0%)	
An 18-year-old male patient went to the dental school for endodontic treatment on tooth #37. After the treatment, the patient couldn't close his mouth, and he got terrified. He started walking with an open jaw, drooling saliva, and desperately trying to close his mouth. Luckily, you saw him in the corridor and assured him by starting the jaw reduction procedure. What did this patient have?	Acute ankylosis of the jaw	0 (0.0%)	2 (10.0%)	>0.999
	Dislocation of the jaw*	3 (100.0%)	17 (85.0%)	
	Disc displacement with reduction	0 (0.0%)	0 (0.0%)	
	Disc displacement without reduction	0 (0.0%)	1 (5.0%)	
A swelling under the tongue, associated with intermittent, acute pain for 1 week, was reported in an 8 year old female patient. Clinical examination revealed a superficial, 5 mm-hard swelling situated near the lingual frenum, which was extremely tender on palpation. There have been no reports of discharge or bleeding in the area. What is the most appropriate diagnosis?	Mucous extravasation cyst	3 (100.0%)	7 (35.0%)	0.207
	Mucous retention cyst	0 (0.0%)	5 (25.0%)	
	Sialolithiasis*	0 (0.0%)	8 (40.0%)	
	Pleomorphic adenoma	0 (0.0%)	0 (0.0%)	
Since she was a teenager, a 34-year-old female attorney has experienced sinus headaches three times per month. The pain, which is typically moderate to severe, is localized to one side of the cheek and feels like pressure or throbbing. She occasionally gets sick to herself, feels like her nose is stuffed, and her nose runs. When the attacks start, she usually lies down in a dark room. Movement and changes in the weather aggravate her headache. Which diagnosis fits her headache the best?	Sinus headache	1 (33.3%)	2 (10.0%)	0.453
	Migraine without aura*	2 (66.7%)	17 (85.0%)	
	Tension type headache	0 (0.0%)	0 (0.0%)	
	Chronic paroxysmal hemicrania	0 (0.0%)	1 (5.0%)	
A 39-year-old woman comes in complaining of pain near her right mandibular first and second premolars. Following mandibular advancement orthognathic surgery, the pain began nearly immediately. There is a moderate amount of pain. A dull, sometimes paroxysmal attack producing quality to the pain is experienced while touching the affected area. An inferior alveolar block was equivocal. Of the following, which ONE is the most likely diagnosis	Atypical odontalgia	0 (0.0%)	3 (15.0%)	>0.999
	Trigeminal neuralgia	0 (0.0%)	0 (0.0%)	
	Traumatic neuroma	0 (0.0%)	4 (20.0%)	
	Traumatic trigeminal neuralgia*	3 (100.0%)	13 (65.0%)	
A strong, quickly developing headache that can last up to 15 minutes is experienced by a 31-year-old female patient. Ipsilateral tears and nasal congestion occur along with the headache. They are dull and throbbing attacks that happen one to four times a day. Whichever diagnosis is most likely?	Chronic paroxysmal hemicrania*	1 (33.3%)	13 (65.0%)	0.589
	Migraine headache	0 (0.0%)	1 (5.0%)	
	Cluster headache	2 (66.7%)	6 (30.0%)	
	Tension type headache	0 (0.0%)	0 (0.0%)	

Table 2 presents the results of the agreement analysis for responses to 20 questions among the subjects under study. The agreement level, measured using the Kappa value, indicates varying degrees of agreement. The agreement among senior dental residents was observed to be weak, with a Kappa value of 0.396, reflecting the challenges in achieving a consensus among residents. In contrast, the agreement among LLMs demonstrated a moderate level, with a Kappa value of 0.622, suggesting a more cohesive alignment in responses among the artificial intelligence models. When comparing residents' responses with those generated by different OpenAI models, including OpenAI, Stablediffusion, and PopAI, the agreement levels were consistently categorized as weak, with Kappa values of 0.402, 0.381, and 0.392, respectively (Table 2).

**Table 2:** Results of the agreement analysis for the responses to 20 questions LLMs: large language models

Parameter	N of raters	Kappa value	Agreement level
Agreement between residents	20	0.396	Weak
Agreement between LLMs	3	0.622	Moderate
Agreement between residents and OpenAI	21	0.402	Weak
Agreement between residents and Stablediffiusion	21	0.381	Weak
Agreement between residents and popAI	21	0.392	Weak

## Discussion

This AI model is very convenient to employ because it is easily accessible to the general public and provides answers in an easily understood language [13]. ChatGPT can offer general guidelines on management plans and treatment processes in situations where healthcare information is insufficient [14]. When considering the use of LLMs in clinical practice, healthcare practitioners need to be cautious of a phenomenon known as "hallucinations," in which LLMs convey false or inaccurate information as fact [15]. According to previous literature, ChatGPT's wide range of services, including customized patient care, scheduling, invoicing, diagnosis, and treatment planning, regular check-ins, and reminders on the patients' dental health, has the potential to transform the dental and healthcare industries [16,17]. ChatGPT demonstrated its extensive understanding of general medicine by passing the US Medical Licensing Exam without the need for formal training [18]. Even in the near future, ChatGPT's performance may increase because the field of generative AI is dynamic and always developing [19].

The results of the current study provide valuable insights into the performance of three LLMs - OpenAI, Stablediffusion, and PopAI - in comparison to the diagnostic abilities of senior dental residents across 20 clinical cases related to oral lesion diagnosis. One striking finding was the substantial variation in performance across specific diagnostic scenarios. Notably, in the case of ectodermal dysplasia, none of the LLMs achieved accurate responses, all erroneously identifying the condition as xeroderma pigmentosum. In contrast, senior dental residents exhibited a significantly higher accuracy rate of 85.0% (p = 0.011). This raises critical questions about the models' ability to accurately differentiate between closely related oral conditions, emphasizing the need for improved specificity in their training datasets. Similarly, the divergence in responses was evident in the management of a large, painful ulcer post-kidney transplant. All three LLMs overwhelmingly favored topical corticosteroid (100%), while 70.0% of senior dental residents correctly opted for intralesional corticosteroid injection (p = 0.022). This discrepancy underscores potential shortcomings in the models' understanding of treatment modalities and the importance of context-specific decision-making.

The median knowledge score for LLMs was 50.0 (IQR: 45.0 to 60.0), with OpenAI exhibiting the highest score (70) and Stablediffusion the lowest (40). In contrast, senior residents demonstrated a higher median score of 65.0 (IQR: 55.0-75.0), with scores ranging from 40 to 90. Despite these variations, statistical analysis did not reveal a significant difference in the overall percent scores between residents and LLMs (p = 0.211).

Implications and limitations

These findings highlight the potential of LLMs in aiding diagnostic processes, but caution must be exercised in specific clinical scenarios where their performance may lag behind human expertise. The observed discrepancies underscore the importance of continuous refinement and augmentation of training datasets to enhance model accuracy, especially in nuanced medical domains. However, this study is not without limitations. The relatively small sample size of 20 clinical cases and the exclusive focus on oral lesion diagnosis may limit the generalizability of the findings. Future research should explore a broader spectrum of medical conditions and expand the dataset to provide a more comprehensive evaluation of LLMs in clinical decision-making.

## Conclusions

In conclusion, this comparative study sheds light on both the potential and limitations of LLMs in the context of oral lesion diagnosis. While LLMs demonstrated comparable overall performance to senior dental residents, specific discrepancies in diagnostic accuracy and treatment recommendations warrant further investigation and refinement of these models to ensure their optimal utility in clinical practice. However, it is crucial to also take into account the inherent limitations and potential risks of the implementation. It is necessary to exercise caution and thoroughly evaluate the advantages and disadvantages. Ultimately, it is important to note that it should not serve as a reason or replacement for not seeking a dentist's expert assessment and diagnosis.

## Appendices

1. A 50-year-old male sought a dental checkup and oral prophylaxis, noting a three-year interval since his last dentist visit. The patient, a daily smoker of 10 cigarettes, reported no other systemic diseases. Examination revealed painless, well-demarcated whitish plaques on the buccal mucosa and both retro-commissures of the lips, which did not detach upon rasping. The patient was uncertain about the duration of these lesions. The clinical features of the white lesions could be diagnosed as?

a) Pseudomembranous candidiasis

b) Erythematous candidiasis

c) Hyperplastic candidiasis

d) Angular cheilitis

e) Herpes simplex virus

2. A 55-year-old female patient with a history of type II diabetes mellitus presented to your clinic with a new-onset (3 months duration) solitary oral pigmented lesion measuring 1 cm × 1 cm on the lower lip. The lesion appears brown to dark brown in color, and its size has remained constant.

a. Perform a biopsy

b. Request a complete blood test

c. Follow up the case and do nothing

d. Take an X-ray to check for any amalgam remnants

3. A 21-year-old male patient presented at your clinic, and during the extra-oral examination, you noted fine, sparse hair, wrinkled, and hyperpigmented periocular skin. This patient is most likely experiencing:

a. Ectodermal dysplasia

b. Xeroderma pigmentosum

c. Darier disease

d. Peutz-Jeghers syndrome

4. A 10-year-old boy, presenting with crusted lips and a history of fever, exhibits red blisters with white centers on his face, hands, and feet. This boy most probably has:

a. Hand-foot and mouth disease

b. Erythema multiforme triggered by food

c. Erythema multiforme triggered by HPV infection

d. Erythema multiforme triggered by HSV infection

5. A 35-year-old female visited your clinic seeking restoration for her missing lower first molars, teeth #36 & #46. During history-taking, she mentioned a three-month complaint of psoriasis, treated with hydrocortisone for affected skin lesions. What oral manifestations would you expect to observe in her condition?

a. Fissure tongue

b. Erythema migrans

c. Hairy tongue

d. Scalloped tongue

6. A 45-year-old female patient with a history of hepatitis C, hypertension, elevated ALT and AST, and blood pressure measured at 135/105 mmHg, presented to the oral medicine clinic with extensive ulcerations, including the buccal mucosa and anterior tongue. Additionally, the lower labial mucosa exhibited white lacy lines with intact mucosa. The most probable diagnosis would be:

a. Mucous membrane pemphigoid

b. Erosive lichen planus

c. Erythema multiforme

d. Pemphigus vulgaris

7. A 42-year-old female patient attending the oral diagnosis course clinic exhibits claw-like fingers and a mouse face. Upon inquiry, she reveals that she has been diagnosed with:

a. Multiple sclerosis

b. Systemic lupus erythematosis

c. Systemic sclerosis

 d. Tuberous sclerosis

8. A 55-year-old female patient, complaining of a toothache, reports a history of splenectomy due to splenomegaly five years ago. You order a complete blood count (CBC) to ensure everything is within normal limits. What would you expect to be elevated in the CBC report?

a. Red blood cells count

b. Platelets count

c. Neutrophils count

d. Eosinophils count

9. In diagnostic anesthesia methods used to localize a source of pain, which of the following conditions would you NOT anticipate a positive response (significant decrease in pain) to a local anesthetic (both injection and topical)?

a. Atypical odontalgia

b. Trigeminal neuralgia

c. Glossopharyngeal neuralgia

d. Traumatic neuroma

10. A 55-year-old female patient, who underwent a kidney transplant three months ago, presents at your clinic with a large painful ulcer in the buccal mucosa. What is the best treatment for this ulcer?

a. Topical lidocaine gel

b. Topical corticosteroid

c. Intralesional corticosteroid injection

d. Systemic corticosteroid.

11. An elderly male patient with a history of Bechet’s disease with long-term steroid use. He came to your clinic to report experiencing pain and swelling on the left side of his face, along with a 2-week period of erythema on his skin. The pain aggravated during mealtime. An intraoral examination revealed pus discharge from the left Stenson's duct. What diagnosis of the following in the top of the list?

a. Sjögren’s syndrome

b. Lymphoma

c. Bacterial sialadenitis 

d. Mucoepidermoid carcinoma

12. Your clinic was visited by a 23-year-old ex-ballet dancer who reported a loud clicking sound on both sides of her TMJs without experiencing any discomfort. The opening of her mouth is 55 mm, with a slight deviation to the right then to the left starting suddenly 16 months ago. Even after mandibular advancement, the examination revealed a clicking sound. The most likely diagnosis in this case is?

a. Disc displacement with reduction

b. Disc displacement without reduction

c. Hypermobility/subluxation of the TMJs

d. Dislocation of the TMJs

13. A 41-year-old female patient who was well-nourished came forward with a main complaint of painful and limited mouth opening. When she opens, her mouth moves towards the right side. One week ago, she reported experiencing a trauma from her teenage son. What is the most likely explanation for this case?

 a. Disc displacement with reduction in the left TMJ

b. Chronic disc displacement without reduction in the right TMJ

c. Acute disc displacement without reduction in the right TMJ

d. Acute disc displacement without reduction in the left TMJ

14. A 31-year-old woman has been reported discomfort in her right cheek for the last two weeks. Vertical opening and excursions are normal. She has a feeling that her bite is off and it hurts when she attempts to bring her teeth together. Her examination revealed a posterior open bite on the right side and premature contact in the left anterior canine area. What is the most likely diagnosis?

a. Acute disc displacement of the right TMJ

b. Capsulitis of the right TMJ

c. Acute disc displacement of the left TMJ

d. Retrodiscitis of the left TMJ

15. A 35-year-old female teacher came to your clinic complaining of severe pain on the left side of her jaw that started three months ago. Systemic osteoarthritis was a significant part of her medical history, and she was diagnosed with it last year. Her pain is intense and has a dull aching quality. The pain is making it difficult for her to open her mouth, even with paracetamol. The best way to control the pain in this case is achieved by?

a. Using non-steroidal anti-inflammatory drugs

b. Increasing the paracetamol dose

c. Starting antibiotic course

d. Giving corticosteroid injections

16. An 18-year-old male patient went to the dental school for endodontic treatment on tooth #37. After the treatment, the patient couldn't close his mouth, and he got terrified. He started walking with an open jaw, drooling saliva, and desperately trying to close his mouth. Luckily, you saw him in the corridor and assured him by starting the jaw reduction procedure. What did this patient have?

a. Acute ankylosis of the jaw

b. Dislocation of the jaw

c. Disc displacement with reduction

d. Disc displacement without reduction

17. A swelling under the tongue, associated with intermittent, acute pain for 1 week, was reported in an 8 year old female patient. Clinical examination revealed a superficial, 5 mm-hard swelling situated near the lingual frenum, which was extremely tender on palpation. There have been no reports of discharge or bleeding in the area. What is the most appropriate diagnosis?

a. Mucous extravasation cyst

b. Mucous retention cyst

c. Sialolithiasis

d. Pleomorphic adenoma

18. Since she was a teenager, a 34-year-old female attorney has experienced sinus headaches three times per month. The pain, which is typically moderate to severe, is localized to one side of the cheek and feels like pressure or throbbing. She occasionally gets sick to herself, feels like her nose is stuffed, and her nose runs. When the attacks start, she usually lies down in a dark room. Movement and changes in the weather aggravate her headache. Which diagnosis fits her headache the best?

a. Sinus headache

b. Migraine without aura 

c. Tension type headache

d. Chronic paroxysmal hemicrania

19. A 39-year-old woman comes in complaining of pain near her right mandibular first and second premolars. Following mandibular advancement orthognathic surgery, the pain began nearly immediately. There is a moderate amount of pain. A dull, sometimes paroxysmal attack- producing quality to the pain is experienced while touching the affected area. An inferior alveolar block was equivocal. Of the following, which ONE is the most likely diagnosis?

a. Atypical odontalgia

b. Trigeminal neuralgia

c. Traumatic neuroma

d. Traumatic trigeminal neuralgia

20. A strong, quickly developing headache that can last up to fifteen minutes is experienced by a 31-year-old female patient. Ipsilateral tears and nasal congestion occur along with the headache. They are dull and throbbing attacks that happen one to four times a day. Whichever diagnosis is most likely:

a. Chronic paroxysmal hemicrania

b. Migraine headache

c. Cluster headache

d. Tension type headache
